# Why do Spanish households separate their e-waste for proper disposal? An econometric analysis

**DOI:** 10.1007/s11356-021-15933-9

**Published:** 2021-09-01

**Authors:** Fernando Arbués, Inmaculada Villanúa

**Affiliations:** 1 Aragon Public Economics Research Group, Institute of Research into Environmental Sciences (IUCA), University of Zaragoza, Violante de Hungría 23, 50009 Zaragoza, Spain; 2 Aragon Growth, Demand and Natural Resources Research Group, Department of Economic Analysis, University of Zaragoza, Gran Vía, 2, 50005 Zaragoza, Spain

**Keywords:** e-waste, Households’ behaviour, Bivariate probit model, Environmental concern, Interaction effects, Sustainable Development Goals

## Abstract

Improving e-waste separate collection rates is of the utmost importance to achieve the environmental targets set in the Sustainable Development Goals. Therefore, this paper aims to identify the factors influencing the intentions of Spanish households to separate their e-waste for proper disposal. To this end, we carry out an econometric analysis revealing that the preoccupation with environmental issues is an endogenous regressor, so a bivariate model is required to avoid inconsistent results. The analysis shows that environmental concern and the origin of the individuals are relevant factors that influence the e-waste separation decision. Additionally, we observe an interaction between age and city size, so the effect of one of these factors on the probability of separating e-waste depends on the other factor. Another important result is that several socio-economic variables and knowledge of environmental issues only indirectly affect attitudes, by way of environmental concern. In summary, this study offers a useful methodology to policymakers who have to deal with e-waste management, allowing them to identify the priority groups on which should be focused, as well as to design specific measures tailored to their characteristics.

## Introduction

The use of electrical equipment and electronic devices has become a typical feature of modern society. A result of the increasing consumption of these products (laptops, monitors, refrigerators, smartphones, etc.) has been that the waste they generate (e-waste) which is considered as the fastest growing waste stream in the world, with 53.6 million tonnes generated in 2019, and it is expected to exceed 74 million tonnes by 2030 (Forti et al. 2020). It is noteworthy that e-waste is a non-homogeneous and complex mixture of diverse hazardous materials that may pose environmental and health risks if not properly collected, treated, and recycled or otherwise disposed of. Moreover, within the paradigm of a circular economy, the separate collection of e-waste is important in terms of recovering valuable and scarce materials, especially copper, and precious metals.^1^

Similar to other developed countries, e-waste is a major concern for European Union (EU) environmental policies focused on reducing its ecological footprint and promoting a circular economy model of economic growth. According to the European Commission (2020), e-waste is growing at 3% to 5 % per year, three times faster than the average waste stream, and EUROSTAT (2021a) shows that only 38.9% is properly recycled. In this context, it should be noted that households are the main source of e-waste in all EU countries (EUROSTAT 2021b). This is the case in Spain, where 87% of e-waste collected in 2018, i.e. 279,100 tons, came from households, while the amount from other sources was 41,522 tons (EUROSTAT 2021b). Furthermore, between 2008 and 2018, 7.172 million tons of electrical and electronic devices for households were put on the market (EUROSTAT 2021b), which indicates the potential e-waste available from Spanish households in the coming years.

To improve the collection and recycling of electrical and electronic equipment, and thus tackle this fast-increasing waste stream, the EU put in place, in 2003, the European Directive on waste electrical and electronic equipment, revised in 2012 (European Union 2012). The Directive requires Member States to ‘adopt appropriate measures to minimize the disposal of e-waste in the form of unsorted municipal waste, to ensure the correct treatment of all collected e-waste, and to achieve a high level of separate collection of e-waste’ (European Union 2012; Art.5 §1). Furthermore, the Directive sets criteria and targets for the collection, treatment, and recovery of e-waste (European Union 2012; Arts. 5, 6 and 7). In this context, meeting the targets set in the Directive requires that Member States ‘adopt appropriate measures so that consumers participate in the collection of e-waste and to encourage them to facilitate the process of re-use, treatment and recovery’ (European Union 2012; Art.14 §3). In Spain, the implementation of this European Directive is based on the Royal Decree on the Disposal of Electrical and Electronic Equipment (Official State Gazette 2015). In accordance with it, the minimum target for e-waste collection from households met in 2019 was 395,422,100.49 kg (MITECO 2020), which represents an increase of 50.65% of the e-waste collected from households in 2017.

Furthermore, increasing levels of e-waste is a major challenge for the achievement of Sustainable Development Goals (SDGs) set out in the 2030 Agenda for Sustainable Development (United Nations 2015), particularly those related to 3.9, 8.3, 8.8, 11.6, 12.4, and 12.5 specific targets (United Nations 2015).

In this context, where the adoption of measures to promote the separate collection of e-waste becomes a priority, the purpose of this study is to identify the behaviours and characteristics of households that influence the participation of Spanish households in the separate disposal of e-waste. Note that the household characteristics and their attitudes towards separating and recycling different kinds of waste is a determinant issue for designing effective measures addressing waste separation and collection as pointed out by many works, such as Aprile and Fiorillo (2019), Dwivedy and Mittal (2013), Saphores et al. (2012), and Zen et al. (2014).

The choice of Spain for our analysis is relevant for two major reasons: first, because Spain is currently showing great interest in the achievement of the SDGs. However, although for some years now, national, regional, and local Spanish authorities have been implementing several measures to promote pro-environmental attitudes (such as e-waste separation), as we have pointed out above, in Spain, a significant improvement of separate e-waste collection rates is needed (European Commission 2019), and secondly, because Spain is located in the Mediterranean Basin, a region highly exposed to significant environmental risks due to climate change (Pausas and Millán 2019), so improving e-waste recovery and recycling rates can generate important benefits by reducing CO_2_ emissions to the atmosphere.

Thus, the objective of this paper is to analyse the participation of Spanish households in e-waste separation and disposal. For this purpose, we estimate a binary model containing two sets of explanatory variables: personal and household characteristics and environmental attitudes, including the preoccupation with environmental issues. Many studies which deal with recycling issues using binary models can be found in the literature. The usual practice in these works is to include the attitudinal factors related with the environment as explanatory variables in the model directly. Nevertheless, including these variables, which are choices made by respondents, directly in the model, an endogeneity problem can arise. Thus, the possible endogeneity of one of the regressors requires specifying a two-equation binary model (bivariate probit model), in order to obtain adequate estimates. In this kind of models, we can distinguish direct and/or indirect effects of the regressors on the dependent variable, the last ones obtained through the endogenous regressor. Additionally, we introduce as regressors the interaction effects between two qualitative factors in the proposed model. Their inclusion allows us to study how a change in one of these factors can be affected by the value of the other.

Hence, the main contributions of our empirical work can be summarized as follows: (1) the estimation of a bivariate probit model with interaction effects to identify the factors determining the correct disposal of e-waste, which has not been used in the context of e-waste studies; (2) since, as we have indicated above, households are an important source of e-waste in most European countries, the results of our analysis can be very useful in exploring ways of reducing the percentage of such waste that households dispose of.

The rest of the paper is organized as follows: Section 2 includes a literature review of previous empirical works focused on household waste management behaviour. Next, Section 3 offers general description of the data and the variables used in the study as well as an explanation of the most important issues of our analytical procedure. The findings of the econometric estimation of the proposed model are presented and discussed in Sections 4 and 5, respectively. In Section 6, we report some concluding remarks.

## Literature review

A significant number of empirical works have addressed the factors influencing the separate collection of waste by households focusing on two groups of variables: socio-economic and demographic variables and psychological determinants of pro-environmental behaviours (environmental awareness, social norms, habits, etc.).^2^

In the first group, as Do Valle et al. (2004) and Saphores et al. (2012) point out, the most commonly considered are income, age, gender, and education level. Other variables, such as household size, the labour status of the individuals, the place of birth of the individuals, the kind of city living, and dwelling characteristics, are also included in several studies with the purpose of detecting the effects of other relevant socio-economic factors on residential waste management (see Table 1). In the second group, the most common variables found in reviewing the literature can be grouped in three categories: general attitudes towards the environment, perceptions related to recycling, and social influences (see Table 1).

**Table 1:** Explanatory variables considered in previous empirical works

Studies	Socio-economic and demographic variables										Psychological variables		
	Income	Age	Gender	Education level	Household size	Labour status	Place of birth	Kind of city	Dwelling characteristics	Marital status	Environmental awareness	Recycling perceptions	Social norms
Afroz et al. (2017)	x	x	x	x							x	x	x
Aprile and Fiorillo (2019)	x	x	x	x	x	x		x	x	x	x	x	
Arbués and Villanúa (2016)	x	x	x	x	x	x	x	x		x	x		
Barr et al. (2001)		x	x				x		x		x	x	x
Budak and Oguz (2008)	x	x		x	x						x	x	
Byrne and O’Regan (2014)		x		x								x	
Cai et al. (2020)	x	x	x	x	x						x	x	
Crociata et al. (2015)		x	x	x							x		
Czajkowski et al. (2017)	x	x	x		x							x	x
Darby and Obara (2005)	x		x									x	
De Feo and De Gisi (2010)		x	x	x		x				x			
Do Valle et al. (2004)	x	x	x	x					x		x	x	x
Dwivedy and Mittal (2013)	x	x	x	x	x			x			x		
Hage et al. (2009)	x	x	x	x					x			x	x
Ferrara and Missios (2005)	x	x		x								x	
Islam et al. (2020)	x	x	x	x	x								
Jafari et al. (2017)	x			x	x					x		x	
Keuschnigg and Kratz (2018)	x	x	x	x	x						x		
Lakhan (2015)	x	x	x	x			x					x	
Lee and Paik (2011)	x	x	x	x	x				x		x	x	
Liu et al. (2020)			x	x					x				
Lo and Liu (2018)	x	x	x	x	x	x						x	
Martinho et al. (2017)		x	x	x	x	x							
Nguyen et al. (2018)		x	x	x				x			x	x	x
Nixon et al. (2009)	x	x		x	x		x		x		x		
Pearson et al. (2012)	x		x	x		x	x					x	
Pérez-Belis et al. (2015b)		x										x	
Perry and Williams (2007)							x						
Purcell and Magette (2010)		x		x					x		x		x
Sidique et al. (2010)	x	x	x	x	x	x				x	x	x	
Saphores et al. (2012)	x	x		x	x		x		x	x	x	x	x
Song et al. (2012)	x	x	x	x	x							x	x
Sorkun (2018)	x	x		x					x				
Tadesse (2009)	x	x	x		x						x		
Vassanadumrongdee and Kittipongvises (2018)	x	x	x	x	x	x			x	x	x	x	x
Vesely and Klöckner (2018)											x	x	x
Wang et al. (2011)	x			x	x				x		x	x	
Xu et al. (2018)	x	x	x	x	x		x					x	x
Yakob et al. (2020)	x	x	x	x		x			x	x	x	x	x
Zen et al. (2014)	x						x		x		x	x	x

Nevertheless, as noted in Arbués and Villanúa (2016) and Hansmann et al. (2006), the relationship observed between these variables and waste separation of households appears to be ambiguous. For example, with regard to income, Aprile and Fiorillo (2019), Dwivedy and Mittal (2013), or Song et al. (2012) show that a higher income increases the recycling probability, while others, such as Do Valle et al. (2004), Saphores et al. (2012), and Wang et al. (2011), suggest that the influence of income on recycling behaviour is reduced. As Hornik et al. (1995) and Arbués and Villanúa (2016) indicate, this is mainly due to these studies being carried out in different geographical areas by researchers who assume different perspectives in their analysis (socio-economic, sociological/psychological, and technical) concentrating their attention on different sets of variables.

From a methodological perspective, it is observed that an assortment of analytical procedures is used to estimate the relationship between disposal behaviour of households and the set of explanatory variables considered in the study. As we can see in Table 2, methods used range from general statistical techniques (e.g. frequency analysis and Chi-squared test) to regression-based methods, which are used in this paper.

**Table 2:** Analytical procedures applied in previous works

Studies	Analytical procedures	
	Regression-based procedures	General statistics procedures
Afroz et al. (2017)	Logistic regression	ANOVA test
Aprile and Fiorillo (2019)	Probit model	
Arbués and Villanúa (2016)	Bivariate probit model	
Barr et al. (2001)		Chi-squared test Cluster analysis Principal component analysis
Budak and Oguz (2008)	Logistic regression	
Byrne and O’Regan (2014)		Frequency analysis Cross tabulation Chi-squared test
Cai et al. (2020)	Binary regression model	
Crociata et al. (2015)	Bivariate probit model	
Czajkowski et al. (2017)	Hybrid multinomial logit Hybrid mixed logit	
Darby and Obara (2005)		Frequency analysis Cross tabulation
De Feo and De Gisi (2010)		Chi-squared test Frequency analysis
Do Valle et al. (2004)	Logistic regression	Principal component analysis
Dwivedy and Mittal (2013)	Logistic regression	
Ferrara and Missios (2005)	Ordered probit model	
Hage et al. (2009)	Ordered probit model	
Islam et al. (2020)	Multinomial logistic regression	Frequency analysis Cross tabulation Chi-squared test
Jafari et al. (2017)	Logistic regression	
Keuschnigg and Kratz (2018)	Binary logistic regressions	
Lakhan (2015)		Unpaired *t*-test
Lee and Paik (2011)	Ordinary least squares	
Liu et al. (2020)		Confirmatory factor analysis Structural equation model
Lo and Liu (2018)	Ordinary least squares	
Martinho et al. (2017)		Frequency analysis Cross tabulation Chi-squared test
Nguyen et al. (2018)		Factor analysis Analysis of moment structures
Nixon et al. (2009)	Rank-ordered logit model	Principal component analysis
Oskamp et al. (1998)	Hierarchical multiple regressions	Principal component analysis
Pearson et al. (2012)	Logistic regression	Chi-squared test Independent group *t*-test Sobel test
Pérez-Belis et al. (2015b)	Ordinal logistic regression	Chi-squared test
Perry and Williams (2007)		Frequency analysis
Purcell and Magette (2010)	Logistic regression	
Sidique et al. (2010)	Poisson regression model	Factor analysis
Song et al. (2012)	Logistic regression	
Sorkun (2018)		Confirmatory factor analysis Structural equation model Bootstrapping approach
Tadesse (2009)	Bivariate probit model	
Vassanadumrongdee and Kittipongvises (2018)	Logistic regression	Factor analysis
Vesely and Klöckner (2018)	Logistic regression	
Wang et al. (2011)	Logistic regression	
Xu et al. (2018)	Multiple regression	One-way ANOVA test
Yakob et al. (2020)		Factor analysis Frequency analysis
Zen et al. (2014)		Factor analysis Analysis discrimination method

Among regression-based procedures, logit binary response models are the more widely used in the empirical works revised. Only few studies estimate probit models (see Table 2). In this paper, as we have indicated above, we estimate a bivariate probit model in order to address the endogeneity that could arise when a single logit or probit model is estimated. This is an approach that only few previous studies, such as Arbués and Villanúa (2016), Crociata et al. (2015), and Tadesse (2009), have considered. In our case, in contrast to these previous works, we include as regressors in the model some interaction variables (the product of the dummies of two different factors). As far as we know, this kind of model (bivariate probit model with interactions) has not been applied to analyse e-waste recycling behaviours.

## Data and methods

### Data

Our dataset is extracted from the ‘Survey on Households and the Environment 2008’ carried out by The Spanish Statistical Institute (INE) on a random sample of 27,832 Spanish households, in each of which a cooperating person was selected. The aim of this survey was analysing environmental attitudes and patterns in Spain (INE 2009).

The data collection method used in this survey was the personal interview, although in some cases the possibility of providing the information online or on a free telephone line was offered (INE 2009).

To ascertain the environmental awareness of individuals, the survey includes a set of preliminary questions with the aim of gathering socio-economic and demographic information (income, age, education, country of birth, etc.) about the individuals selected. Regarding environmental awareness, the respondents were asked about environmental concerns, detection of environmental problems, and knowledge of environmental campaigns. Furthermore, to ascertain the attitudes of individuals about their waste management, they were also asked about the sorting and deposit at specific collection points of a set of wastes, among which were e-waste^3^.

The files containing the microdata of this survey are available at INE (2010).

### Variable specification

As we have indicated above, the dependent variable of our empirical study, that we call *Separate*, is a binary variable. It takes value 1 for households that collect separately their e-waste and 0 otherwise. To explain this variable, we have considered two sets of factors: personal and household characteristics and environmental attitudes. These potential explanatory factors are summarized in Table 3. Most of them are qualitative, so they must be quantified by using dummy variables. Table 3 shows both the factors (first column) and the corresponding quantitative variables (second column), together with its description (third column). Some of the factors have more than two categories, so we need more than one dummy to capture its effect.^4^ It is noteworthy that dummy variables corresponding to the *Age* factor are not mutually exclusive and can equal 1 jointly if people from different ranges of age lives in a household.

**Table 3:** Selected explanatory variables

Dependent variable: *Separate* (collect separately their e-waste= 1, otherwise = 0)		
**Explanatory variables**		
**Set 1: personal and household characteristics**		
***Factor***	***Variable***	***Description***
*Age*	*Until20*	There are members who are until 20 years (yes = 1; no = 0)
	*M20to30*	There are members who are from 20 to 30 years (yes = 1; no = 0)
	*M30to45*	Household with members who are from 30 to 45 years (yes = 1; no = 0)
	*M45to60*	There are members who are from 45 to 60 years (yes = 1; no = 0)
	*M60to70*	There are members who are from 60 to 70 years (yes = 1; no = 0)
	*More70*	There are members who are more than 70 years (yes = 1; no = 0)
*Nmembers*	*Nmembers*	Number of persons living in the household
*Married*	*Married*	Marital status (married =1; otherwise = 0)
*Earnings*	*Hearnings*	Monthly earnings more than 2700€ (yes = 1; no = 0)
	*Mearnings*	Monthly earnings between 1101€ and 2700€ (yes = 1; no = 0)
	*Learnings*	Low earnings (monthly earnings less than 1101€, otherwise = 0)
*Origin*	*Origin*	The origin of the respondent (people born in Spain = 1; 0 = otherwise)
*Education*	*Beatt*	Basic educational attainment (yes = 1; no = 0)
	*Meatt*	Medium educational attainment (yes = 1; no = 0)
	*Heatt*	High educational attainment (yes = 1; no = 0)
*Relationship with labour market*	*Occ*	Occupied (yes = 1; no = 0)
	*Unocc*	Unoccupied (yes = 1; no = 0)
	*Pensioner*	Pensioner (yes = 1; no = 0)
	*Student*	Student (yes = 1; no = 0)
*City Size*	*Large_city*	Provincial capitals and cities with 100,000 or more inhabitants (yes = 1; no = 0)
	*Med_city*	Cities from 20,000 to 100,000 inhabitants (yes = 1; no = 0)
	*Small_city*	Cities with less than 20,000 inhabitants (yes = 1; no = 0)
*Gender*	*Gender*	Gender of respondent (man = 1; woman= 0)
**Set 2: Environmental attitudes**		
***Factor***	***Variable***	***Description***
*Preoc_envir*	*Preoc_envir*	Preoccupation with environmental issues (yes = 1; no = 0)
*Awar_camp*	*Awar_camp*	Knowledge of some environmental awareness campaign (yes = 1; no = 0)
*Percep*	*Percep*	Perception of environmental troubles in his/her neighbourhood in the last year (yes =1; no = 0)

In Table 4, the summary statistics of the variables are shown. Most respondents were born in Spain, were working, and had the basic educational attainment. The information concerning household earnings suggest that households belong mostly in the middle class (54.7%). Regarding age, a significant percentage of households had some member in the age range between 30 and 60 years old. The rest of the socio-economic variables (gender, city size, and marital status) were evenly distributed, and the average household size was 2.596. The information corresponding to the environmental attitudes show that the majority of households (78.3%) responded affirmatively to the question on whether they were concerned about the environment. Moreover, 61.5% of the respondents stated that they had knowledge of any environmental awareness campaign (saving water, energy, recycling, etc.) in the last year. Finally, among the sample households, only 26% reported that they had some environmental problem (pollution, noise, bad smells, etc.) in their surroundings.

**Table 4:** Descriptive statistics of the variables

**Set 1: personal and household characteristics**			
***Factor***	***Variable***	***Mean***	***Std. Dev.***
*Age*	*Until20*	0.344	0.475
	*M20to30*	0.253	0.434
	*M30to45*	0.417	0.493
	*M45to60*	0.355	0.478
	*M60to70*	0.199	0.399
	*More70*	0.258	0.438
*Nmembers*	*Nmembers*	2.596	1.234
*Married*	*Married*	0.559	0.496
*Earnings*	*Hearnings*	0.123	0.328
	*Mearnings*	0.547	0.498
	*Learnings*	0.330	0.470
*Origin*	*Origin*	0.243	0.429
*Education*	*Beatt*	0.574	0.494
	*Meatt*	0.268	0.443
	*Heatt*	0.157	0.364
*Relationship with labour market*	*Occ*	0.478	0.499
	*Unocc*	0.058	0.233
	*Pensioner*	0.253	0.435
	*Student*	0.037	0.188
*City Size*	*Large_city*	0.378	0.485
	*Med_city*	0.378	0.485
	*Small_city*	0.243	0.429
*Gender*	*Gender*	0.453	0.498
**Set 2: environmental attitudes**			
***Factor***	***Variable***		
*Preoc_envir*	*Preoc_envir*	0.783	0.412
*Awar_camp*	*Awar_camp*	0.615	0.487
*Percep*	*Percep*	0.260	0.439

### Analytical procedure

In order to explain the dependent binary variable *Separate*, the binary model that we specify has the general expression:

1 $$ p=F\left(\mathbf{x}\upbeta \right) $$

being*p*the probability of separating e-waste, that is, *p*= *pr*(*Separate* = 1), **x** the regressor vector, and **β** the parameter vector. Moreover, *F* is a cumulative distribution function, which can be normal, if the binary model is a probit model *p* = Φ(**xβ**), or logistic, if we specify a logit model *p* = Λ(**xβ**).^5^ Expression (1) has an underlying latent model that can be written as:

2 $$ {Y}^{\ast }=\mathbf{x}\boldsymbol{\upbeta } +u $$

where Y* is a non-observable variable representing level of utility or satisfaction and *u* is the error term, whose distribution normal or logistic leads to the probit and logit model above mentioned. Thus, the binary variable *Separate* adopts value 1 if Y*>0 and value 0 if Y*<0.

The adequate estimation procedure for these models is the maximum likelihood (ML), which requires solving a nonlinear equation system, through a nonlinear optimization algorithm. The ML procedure provides consistent estimates of **β **unless one (or more) of the regressors is not exogenous.

The coefficients included in **β** only provide the sign of the change in probability *p* under changes in the explanatory factors. Therefore, in order to quantify these changes, it is necessary to calculate the marginal effects as *∂p*/*∂X*_*j*_ or Δ*p*/Δ*X*_*j*_ for continuous and discrete regressors, respectively.

For a generic dummy variable *D*_*j*_, with estimated coefficient ${\hat{\beta}}_{D_j}$, and assuming a probit model (*F*=Φ), the corresponding marginal effect from changing *D*_*j*_from zero to one can be calculated as:

3 $$ \Delta \hat{p}/\Delta {D}_j= pr\left({Y}_1=1\left|{D}_j=1\Big)\right.- pr\right({Y}_1=1\left|{D}_j=0\Big)\right.=\Phi \left(\mathbf{x}\hat{\boldsymbol{\upbeta}}-{D}_j\cdot {\hat{\beta}}_{D_j}+{\hat{\beta}}_{D_j}\right)-\Phi \left(\mathbf{x}\hat{\boldsymbol{\upbeta}}-{D}_j\cdot {\hat{\beta}}_{D_j}\right) $$

On the other hand, for a continuous variable, *X*_*j*_, this effect would be obtained as:

4 $$ \partial \hat{p}/\partial {X}_j=\left(\partial \mathbf{x}\hat{\boldsymbol{\upbeta}}/\partial {X}_j\right)\phi \left(\mathbf{x}\hat{\boldsymbol{\upbeta}}\right) $$

Given that **xβ** is linear with respect to *X*_*j*_, then $\left(\partial \mathbf{x}\hat{\boldsymbol{\upbeta}}/\partial {X}_j\right)={\hat{\beta}}_j$, so $\partial \hat{p}/\partial {X}_j={\hat{\beta}}_j\phi \left(\mathbf{x}\hat{\boldsymbol{\upbeta}}\right)$.

In both discrete and continuous cases, each marginal effect depends on all the estimated coefficients and all the regressors of the model, so their value is different for every observation. In order to obtain an aggregate measure, the average of these marginal effects should be calculated.

We have indicated above that the regressor *Preoc_envir* could be an endogenous binary variable, and we need to be sure of it in order to estimate the model adequately. Thus, if this regressor is endogenous, the probit estimates described above will be inconsistent, so we should estimate a bivariate probit model which, following Wooldridge (2002; p.477), can be written as follows:

5 $$ {Y}_1=1\left[\mathbf{x}\boldsymbol{\upbeta } +{u}_1>0\right]\kern1em $$

6 $$ {Y}_2=1\left[\mathbf{z}\boldsymbol{\upgamma } +{u}_2>0\right] $$

where *Y*_1_ and *Y*_2_ are the binary variables previously named as *Separate* and *Preoc_envir*, respectively; the regressor vector of Eq. (5) includes the endogenous *Y*_2_ together with the exogenous **z**_1_, that is, **x** = (**z**_1_ *Y*_2_); **z** is the regressor vector of (6); the parameter vectors in (5) and (6) are **β** and **γ**, where **β** '  = (**δ** *α*_1_); and finally, the error vector (*u*_1_, *u*_2_) is distributed as bivariate normal with mean zero, both *u*_1_ and *u*_2_ have unit variance, and the correlation between them is*ρ*. In Eqs. (5) and (6), 1[⋅] denotes an index, with value 1 if the condition in the brackets is satisfied and zero otherwise. Both conditions correspond to the respective latent equations $$ {Y}_1^{\ast }={\mathbf{z}}_{\mathbf{1}}\boldsymbol{\updelta} +{\alpha}_1{Y}_2+{u}_1 $$ and $$ {Y}_2^{\ast }=\mathbf{z}\boldsymbol{\upgamma } +{u}_2 $$$$ {\mathrm{Y}}_2^{\ast }={\mathrm{z}}_2{\updelta}_2+{\mathrm{v}}_2 $$, being $$ {Y}_1^{\ast } $$ and $$ {Y}_2^{\ast } $$ two latent (non-observable) variables, which can be understood as the level of satisfaction of the option numbered with 1 values in *Y*_1_ and *Y*_2_, respectively. The variables included in vectors **x** and **z** of Eqs. (5)–(6) are shown in Table 5.

**Table 5:** Variables of the bivariate probit model (5)–(6)

**Eq. (****5****)**		**Eq. (****6****)**
**Vector x**		**Vector z**
**z**_**1**_	**Y**_**2**_	
*Learnings*	*Preoc_envir*	*Learnings*
*Origin*		*Beatt*
*Occ*		*Large_ciy*
*Large_ciy*		*Gender*
*Nmembers*		*Married*
*M20to30*		*Origin*
*M45to60*		*Heatt*
*M60to70*		*Awar_camp*
*More70*		Percep
		*M20to30*
		*M45to60*
		*M60to70*
		*More70*

Although our interest is focused on the probability of separating e-waste, if *Y*_2_ is endogenous, we must estimate jointly (5) and (6). With this aim, we construct the log-likelihood by combining the probability of the four outcomes of (*Y*_1_, *Y*_2_) : *P*(*Y*_1_ = 1|*Y*_2_ = 1, **z**), *P*(*Y*_1_ = 1|*Y*_2_ = 0, **z**), *P*(*Y*_1_ = 0|*Y*_2_ = 1, **z**), and *P*(*Y*_1_ = 0|*Y*_2_ = 0, **z**)^6^. Once we estimate (5)–(6) by ML, we test whether the correlation coefficient between the error terms of both equations, which we have called *ρ*, is, in fact, zero. The ML estimation of the bivariate model (5)(6) provides the estimate $\hat{\rho}$. If we reject *H*_0_ : *ρ* = 0, then we calculate the estimated probabilities of *Y*_1_ = 1 from the estimates ${\hat{\boldsymbol{\updelta}}}_{\mathbf{1}}$ and ${\hat{\alpha}}_1$ in Eq. (5) of the bivariate probit model. However, if we do not reject*H*_0_, we can conclude that *Y*_2_ is not an endogenous variable, so we must use the result of the estimation of the one-equation probit model *p* = *F*(**xβ**).

Moreover, it should be noted that if the *Preoc_envir* variable were an endogenous binary regressor in Eq. (5) of the bivariate probit model, its marginal effect would be obtained directly from the bivariate estimation procedure that following the notation established above is *P*(*Y*_1_ = 1|*Y*_2_ = 1) − *P*(*Y*_1_ = 1|*Y*_2_ = 0).

## Results

### The estimation of the model

The results of the bivariate probit estimation of Eqs. (5) and (6) are presented in Table 6. Note that preliminary estimations allowed grouping some of the categories initially defined for *Earnings* (*Hearnings* with *Mearnings*), *Relationship with labour market* (*Unocc* with *Pensioner* and with *Student*), and *City Size* (*Small_City* with *Med_City*) reducing in this way the number of these kind of variables included in the final model.

**Table 6:** Bivariate probit estimation results

**Eq. (1)** **Dependent variable:** ***Separate***		**Eq. (2)** **Dependent variable:** ***Preoc_envir***	
**Regressors (z**_**1**_**)**	**Coefficient value**	**Regressors (z)**	**Coefficient value**
***Const***	–0.7972***	***Const***	0.3897***
***Preoc_envir***	0.8679***	***Learnings***	–0.0673***
***Learnings***	–0.2140***	***Beatt***	–0.1866***
***Origin***	0.5081***	***Large_ciy***	0.0780***
***Occ***	–0.0439*	***Gender***	–0.1403***
***Large_ciy***	–0.0439**	***Married***	0.1386***
***Nmembers***	0.0522***	***Origin***	0.1477***
*M20to30*	–0.0801***	***Heatt***	0.1055***
*M45to60*	0.1991***	*Awar_camp*	0.5051***
*M60to70*	0.1500***	Percep	0.4034***
*More70*	0.1075***	*M20to30*	–0.0446*
		*M45to60*	0.0833***
		*M60to70*	0.0648**
		*More70*	–0.1866***

*, **, and *** indicate statistical significance at the 10%, 5%, and 1% level, respectively

Moreover, the results of the independence test between the error terms of Eqs. (5) and (6) are presented in Table 7.

**Table 7:** Test of independence of the two equations in the bivariate model

$\hat{\rho}$	–0.3509694
Hypotheses	$$ {\displaystyle \begin{array}{l}{H}_0:\rho =0\\ {}{H}_A:\rho \ne 0\end{array}} $$
Wald test value	41.1827
Distribution	${\chi}_1^2$
*p* value	0.0000

The Wald test, which is distributed as ${\chi}_1^2$, has a value of 41.1827. The corresponding *p* value (zero) implies that the null hypothesis *H*_0_ : *ρ* = 0 is rejected, so both error terms are non-independent. This result allows us to conclude that we must jointly estimate both Eqs. (5) and (6) as a bivariate probit model, in order to obtain consistent estimates.

If we observe the left side of Table 6, corresponding to Eq. (5), we can see the estimated parameters of factors that have a direct effect on the decision of households to deposit their e-waste at collection points. The coefficients of *Preoc_envir* and *Origin* are both positive, and they have the highest values. The estimated parameter of *Nmembers* is also positive, although its value is lower.

On the other hand, the estimated parameters corresponding to the variables *Large_city*, *Learnings*, and *Occ* are negative. Thus, respondents living in a large city have a probability of separating e-waste lower than that of respondents living in medium/small cities (control group). With respect to the *Learnings* variable, individuals with low incomes have a lower probability of recycling than those with medium or high levels of income (control group). Analogously, working individuals have a smaller probability of separating e-waste, with respect to people in other labour situations (control group including student, unoccupied, or pensioner).

Finally, regarding the age interval dummies, we observe a negative sign for *M20to30*, indicating that the presence of people between 20 and 30 years old in the household reduces the probability of separating e-waste against the households where there are no people of this age range. However, the presence of people from the other age ranges considered increases this probability, as indicates the positive sign of their estimated coefficients.

Focusing now on the right side of Table 6, corresponding to Eq. (6) whose dependent variable is *Preoc_envir*, we can see the factors that have a direct effect on the probability of having environmental concern and also an indirect effect on the probability of separating e-waste, which is our main interest. In this way, it should be noted that there are four variables in Eq. (6) (*Learnings*, *Large_city*, *Origin*, and the interval age dummies) that also appeared in Eq. (5). In all of them, the sign of both direct and indirect effects is the same so the environmental concern of individuals reinforces the effect of these socio-economic variables on separating e-waste.

Furthermore, the variables *married*, *gender*, and basic and high education (*Beatt* and *Heatt*), as well as those that reflect the influence of knowledge of environmental issues (*Awar_camp* and *Percep*), have only indirect effects. In this way, the sign of the estimated coefficients of these variables shows how they contribute to sorting e-waste intentions through their environmental concern.

As can be seen in Table 6, women and married people show a higher tendency to separate their e-waste than men and single people, respectively.

As we have just seen, the estimated coefficient of every regressor allows us to know the direction of the change in the probability when a regressor changes. However, it is possible that the effect of the age of members of the household on the probability differs depending on the level of earnings, the labour situation, and so on. In order to take this into account, we have defined some interaction variables (the product of the dummies of two different factors) to be included as regressors in Eq. (5). Specifically, interactions between the age interval of members in the household and the city size provide significant results. The results of the estimation of this bivariate probit model with interactions are shown in Table 8.

**Table 8:** Estimation of a bivariate probit model with interaction variables

**Eq. (1). Dependent variable:** ***Separate***			**Eq. (2). Dependent variable:** ***Preoc_envir***		
**Coeficient** **name**	**Regressors (z**_**1**_**)**	**Coefficient** **value**	**Coeficient** **name**	**Regressors (z)**	**Coefficient** **value**
*α*_1_	***Preoc_envir***	0.8686***	*γ*_0_	***Const***	0.3895***
*δ*_0_	***Const***	–0.7725***	*γ*_1_	***Learnings***	–0.0674***
*δ*_1_	***Learnings***	–0.2102***	*γ*_2_	***Beatt***	–0.1863***
*δ*_2_	***Origin***	0.5078***	*γ*_3_	***Heatt***	0.1061***
*δ*_3_	***Occ***	–0.0415*	*γ*_4_	***Large_ciy***	0.0776***
*δ*_4_	***Large_ciy***	–0.1173***	*γ*_5_	***Gender***	–0.1403***
*δ*_5_	***Nmembers***	0.0524***	*γ*_6_	***Married***	0.1383***
*δ*_6_	***M20to30***	–0.0689**	*γ*_7_	***Origin***	0.1476***
*δ*_7_	***M45to60***	0.1633***	*γ*_8_	***Awar_camp***	0.5049***
*δ*_8_	***M60to70***	0.1029***	*γ*_9_	***Percep***	0.4037***
*δ*_9_	***More70***	0.0724**	*γ*_10_	***M20to30***	–0.0447*
*δ*_10_	***Large_M20to30***	–0.0306	*γ*_11_	***M45to60***	0.0838***
*δ*_11_	***Large_M45to60***	0.0951**	*γ*_12_	***M60to70***	0.0656**
*δ*_12_	***Large_M60to70***	0.1293**	*γ*_13_	***More70***	–0.1861***
*δ*_13_	***Large_More70***	0.0966**			
Wald test (for *H*_*0*_: *ρ* = 0) = 41.248, *p* value=0.0					

*, **, and *** indicate statistical significance at the 10%, 5%, and 1% level, respectively

As we can see in Table 8, the Wald test indicates that the errors of Eqs. (5) and (6) are not independent, so the bivariate probit model procedure is adequate.

Table 8 shows that parameters of all interaction terms (*δ*_11_, *δ*_12_, and*δ*_13_) are significant at 5% and 10%, except that of *Large_M20to30* (*δ*_10_). This non-significance means that the city size (large or small) does not affect the change in the probability of separating e-waste between households with individuals from 20 to 30 years old and those without them. Analogously, to have or not to have individuals in this age range has no influence on the effect of city size on the probability of separating. This result is different for the other age ranges, as we can derive from the statistical significance of*δ*_11_, *δ*_12_, and*δ*_13_.

Regarding the other explanatory factors considered in the model, we see that *Occ* is only significant at 10%, while the others are significant at 1% and 5%. The sign of the coefficients in Table 8 holds with respect to those of the previous estimation without interactions (Table 6). The estimated magnitude for parameters of variables not affected by the interaction is also held.

### The marginal effects

The average marginal effects corresponding to the bivariate probit model without interactions (see Table 6) are presented in Table 9. It is noteworthy that *Preoc_envir* has the largest value among the marginal effects (0.3381), followed by *Origin* (0.1789).

**Table 9:** Average marginal effects of bivariate probit model without interaction variables

**Eq. (1).** **Dependent variable:** ***Separate***	
**Regressors(z**_**1**_**)**	**Coefficient value**
***Preoc_envir***	0.3381***
***Learnings***	–0.0712***
***Origin***	0.1789***
***Occ***	–0.0142*
***Large_ciy***	–0.0143**
***Nmembers***	0.0169***
***M20to30***	–0.0263***
***M45to60***	0.0642***
***M60to70***	0.0478***
***More70***	0.0345***

*, **, and *** indicate statistical significance at the 10%, 5%, and 1% level, respectively

Then, in the estimation of Eq. (5), four interaction terms were introduced: (*Large* x *M20to30*), (*Large* x *M45to60*), (*Large* x *M60to70*), and (*Large* x *More70*). As we can see in Table 10, from these four interaction terms, it is possible to define sixteen categories based on age range of household members and city size.

**Table 10:** Name of the groups defined through interaction variable

	**Age interval A**: 20–30 years		**Age interval B**: 45–60 years		**Age interval C**: 60–70 years		**Age interval D**: more than 70	
	With	Without	With	Without	With	Without	With	Without
Large	GL1_A	GL0_A	GL1_B	GL0_B	GL1_C	GL0_C	GL1_D	GL0_D
Small	GS1_A	GS0_A	GS1_B	GS0_B	GS1_C	GS0_C	GS1_D	GS0_D

In Tables 11, 12, and 13, the marginal effects for the model with interaction variables are presented. Note that the calculation of those corresponding to regressors with interactions is quite different from that described in (3). Details about the way of obtaining them are in Appendix 1. Specifically, Table 11 shows the average marginal effects of the city size for every age range and Table 12 the average marginal effects of the age intervals for large cities and for small cities.

**Table 11:** Average marginal effects of city size

	**20–30**	**45**–**60**	**60**–**70**	**More than 70**
**Pr(GL1)-Pr(GS1)**	–0.0221737***	0.005785	0.0184338	0.0083113
**Pr(GL0)-Pr(GS0)**	–0.0116819***	–0.0256195***	–0.022623***	–0.0226978***

*, **, and *** indicate statistical significance at the 10%, 5%, and 1% level, respectively

**Table 12:** Average marginal effects of age ranges

	**20–30**	**45–60**	**60–70**	**More than 70**
**Pr(GL1)-Pr(GL0)**	–0.032939***	0.0838692***	0.0738832***	0.0541919***
**Pr(GS1)-Pr(GS0)**	–0.0224472**	0.0524647***	0.0328264***	0.0231828**

*, **, and *** indicate statistical significance at the 10%, 5%, and 1% level, respectively

**Table 13:** Average marginal effects with interaction variables

**Eq. (1). Dependent variable:** ***Separate***	
**Regressors (z**_**1**_**)**	**Coefficient value**
***Preoc_envir***	0.3381***
***Learnings***	–0.0698***
***Origin***	0.1787***
***Occ***	–0.0135*
***Nmembers***	0.0170***

*, **, and *** indicate statistical significance at the 10%, 5%, and 1% level, respectively

Focusing on Table 11, the first row shows the change in the probability of separating e-waste between large and small cities, when there are household members in a given age range. Thus, if the household includes members belonging to the age range 20–30 years old, the probability of separating e-waste in a large city decreases by 0.022 in comparison with a medium to small city. There are almost no differences in probability between large and small cities if there are members in the age intervals 45–60 years old (0.0057) and more than 70 years old (a change of 0.0083). When there are individuals between 60 and 70 years old, the probability slightly increases (a change of 0.018).

The second row of Table 11 provides the changes in probability between large and small cities when there are no individuals in a given age interval. We see that in large cities, this probability is lower than that in small cities, whatever the age interval considered.

In the model without interactions, the probability of separating e-waste in a large city was lower by 0.014 than in a small city, as we can see in Table 9. Now, in Table 11, we can see absolute values lower and higher than 0.014, being some of them positive.

The two rows of Table 12 give the change in probability when we compare the households with and without members from a given age range. As we can see in Table 9, without interaction between age and city size, the effects were –0.0263, 0.0642, 0.0478, and 0.0345 for the four intervals, respectively. Now, with interactions, the sign is held, but the absolute value is larger when the household is in a large city, indicating that the effect of the age of members in the household on the probability of recycling e-waste depends on the city size.

It should be noted that having individuals from 20 to 30 years old in the household (i.e. those who consume most electrical and electronic devices) reduces the probability of separating e-waste in both large and small cities. Another noteworthy result is that the largest increase in the probability of separating e-waste occurs in those households in large cities where some of its members are in the age ranges 45 to 60 and 60 to 70. Specifically, this probability increases by 0.083 and 0.073 with respect to households in large cities where there is no member in these age ranges. In small cities, these increases in the probability of separating e-waste in these two age ranges are lower (0.052 and 0.032, respectively).

Hence, including interaction terms can provide more reliable estimations for the effects of the explanatory variables. To complete this analysis, Table 13 shows the average marginal effects corresponding to the regressors included in the estimated model with interactions (Table 8), but not implied in the interactions. The results do not change with respect to Table 9, as expected.

## Discussion

In this study, we have observed that there are two relevant factors in explaining the decision of households to collect separately their e-waste for proper disposal: the preoccupation with environmental issues and the origin of the people.

Regarding the first variable (*Preoc_envir*), the econometric estimation reveals that it positively affects the attitudes of households towards e-waste separation and collection. It should be noted that this result is in line with the bulk of the prior research concluding that a pro-environmental concern encourages households to manage their e-waste appropriately (e.g. Arbués and Villanúa 2016; Keuschnigg and Kratz 2018). Therefore, the relevance of environmental concern in the intention to properly disposal e-waste points to the crucial role that environmental education must play in promoting proper e-waste management in the households. It is noteworthy that this result supports those of Pérez-Belis et al. (2015b) who concluded that in order to avoid unsorted disposal of electric and electronic toys, it was necessary to train and provide information to consumers about how to manage this kind of e-waste properly.

Thus, it will be very important to reinforce the environmental awareness programmes related to SDGs promotion campaigns, periodically designing campaigns of short duration aimed at different education levels (from preschool to post-secondary). In addition, emphasis should be placed on introducing into the contents of science subjects (for example, in the Spanish secondary education level curriculum, there is a subject labelled scientific culture) several topics that reinforce concern about the environmental problems of hazardous waste in general and e-waste in particular. In summary, environmental education should be a lifelong learning process to promote a proactive environmental citizenship. In this context, the collaboration between environmental authorities (national, regional or local), the educational system, producers and companies (as remark Pérez-Belis et al. 2015b), communication media, and even the so-called influencers, who have a great capacity to impact their followers on social networks, should be enhanced to ensure engagement of households in e-waste separate collection.

Focusing on the second variable (*Origin*), the positive sign of its estimated parameter indicates, as well as Lakhan (2015) and Zen et al. (2014), that there are discrepancies in attitudes of households towards their e-waste management depending on the origin of their members. Specifically, this positive sign means that individuals born in Spain tend to separate their e-waste more often than those born in other countries. Furthermore, this result suggests, in line with Pearson et al. (2012) and Perry and Williams (2007), that the practice of separating e-waste is influenced by the perception of the problems related to e-waste in their origin countries and by their consumption patterns (i.e. people from developing countries and some regions of Eastern Europe tend to practise “informal” recycling and reuse). It will therefore be important to spread measures in different languages since many of the people born out of Spain *who do not come from Latin America* are often not fluent in the Spanish language. Additionally, given the different levels of environmental concern among nations around the world, the migrant social networks (diaspora organizations, nongovernmental organizations, religious and cultural organizations, and so on) should play an important role in getting the message about the importance of recycling across to these communities.

A further factor considered in the study with a positive estimated parameter is the household size (*Nmembers*). Note that this result is consistent with many previous empirical works such as that of Martinho et al. (2017), Pérez-Belis et al. (2015b), or Sidique et al. (2010), among others. Further, the value of this estimated parameter is quite low, so the probability of households properly managing the e-waste increases slightly with their size. Its shows consistency with Sidique et al. (2010) and Lo and Liu (2018) which indicate that the effects of household size on proper waste management are limited.

On the other hand, we can also observe in the study that *Large_city*, *Learnings*, and *Occ* have negative estimated coefficients. As we have indicated above, in the case of *Large_city*, this negative relationship means that respondents living in a large city have a probability of separating e-waste lower than that of respondents living in a medium or small city. Although several prior studies find a positive sign for this variable (e.g. Aprile and Fiorillo 2019; Dwivedy and Mittal 2013), this result supports the findings that distance to collection points is a barrier to recycling obtained by Hage et al. (2009) and Sorkun (2018), among others. It is noteworthy that in Spain, the collection points to deposit e-waste are usually on industrial estates located outside the city, so that separate disposal of e-waste implies storage, time, and transport costs for individuals. The larger the city, and the greater the distance to the collection point, these costs grow, and recycling efforts decrease.

Concerning the *Learnings* variable, its negative estimated coefficient implies that the individuals with low incomes have a lower probability of properly disposing their e-waste than those with higher levels of income. This negative relationship is similar to that observed in many preceding works, such as those of Song et al. (2012) and Zen et al. (2014), and may be related to the fact that informal recycling sometimes can be a source of additional income for these households. Furthermore, this result is in line with the positive sign of the estimated coefficient of the variable *Origin*. In Spain, according to INE (2020), the average income per person of foreign residents is lower than that of the Spanish nationals (a 47.78% below in the case of non- EU foreigners and 35.44% below in the case of UE foreigners).

On other hand, the negative estimated coefficient of *Occ* indicates that working individuals have a smaller probability to dispose of their e-waste correctly, than people who are in other labour situations. This is a piece of evidence that few prior empirical works have found (Sidique et al. 2010; Yakob et al. 2020). However, this negative relationship is consistent with the idea implicit in the estimated coefficient for *Large_city*, which pointed out that time availability is a relevant factor in recycling activities. Thus, working people are probably to have more time constraints than unemployed, students, or retired people to conduct recycling activities on a regular basis and even for disposing their e-waste at collection points (e.g. incompatibility between their working hours and the opening hours of the collection points).

Regarding age, the relations estimated between age intervals and recycling behaviour are in parallel with previous studies (Sidique et al. 2010; Sorkun 2018). Specifically, our study shows that the presence of people between 20 and 30 years old in the household reduces the probability of properly managing their e-waste against the households where there are no people of this age range. However, the presence of people from the other age ranges considered increases this probability, as indicates the positive sign of their estimated coefficients. This result is consistent with the fact that households with young members tend to have more electronic devices (smartphones, tablets, laptops, etc.) than households without them. Furthermore, as remark Martinho et al. (2017), it is common for young people to keep their old devices as an alternative in case of breakdown or loss of the new one. Sometimes, the lack of knowledge between young people about where to deliver the old devices (Martinho et al. 2017) and the inconvenience of having to go to a specific location are other factors influencing this result.

At this point, it should be noted that the results obtained from the interaction between the age of the individuals and the size of the city show the need to focus greater attention on people between 20 and 30 years old living in large Spanish cities. This calls attention to the importance of designing campaigns adapted to the digital communication and of using the trending social networks among young people (e.g. YouTube, Twitch, TikTok, or Instagram) as priority channels for spreading messages about the importance of properly managing e-waste. It is noteworthy that, as Pérez-Belis et al. (2015b) show, the way in which campaigns convey the environmental information is key to their success.

Similar to previous empirical works, such as Aprile and Fiorillo (2019), De Feo and De Gisi (2010), and Pearson et al. (2012), our estimation finds that women and married people are more prone to properly manage their e-waste than men and single people, respectively. It is noteworthy that this positive relationship between these variables and recycling behaviour may be explained by the fact that in Spain, according to INE (2011), women dedicate more time than men to both household tasks (such as sorting waste) and volunteer activities (including pro-environmental activities). Regarding the effect of the educational variables considered, the sign of the coefficients (negative for basic education and positive for high education) indicates that the higher the level of education, the more likely for the households to separate their e-waste. This finding corroborates the importance of education to commit people to adopting proactive environmental attitudes, such as proper management of their e-waste (Pérez-Belis et al. 2015b). This positive correlation between high education and recycling behaviour is consistent with most previous papers, as those of Aprile and Fiorillo (2019), Liu et al. (2020), Sidique et al. (2010), and Sorkun (2018), among others.

Finally, note that most of the results obtained in our study indicate that the time and knowledge of how to access the collection points are a major impediment to adequately separate e-waste. For this reason, it is necessary to implement measures aimed at improving the collection point networks (i.e. extending their opening hours, installing e-waste drop-off points in shopping centres and retail stores, or increasing the frequency and the number of mobile e-waste collection points) and the information about where and how households can dispose of their e-waste to be recycled.

## Concluding remarks

The purpose of this paper is to analyse the factors influencing the intention of Spanish households to separate their e-waste for proper disposal. Our analysis shows that environmental concern should be considered as an endogenous binary variable, rather than exogenous, when analysing the determinants of behaviour towards separate collection of e-waste. This is a very relevant result that has clear implications for policymakers, since it indicates that to properly analyse the environmental behaviours of households such as e-waste separate collection, it is necessary to estimate a two-equation binary model, rather than the commonly used one-equation probit, because the latter obtains inconsistent estimates.

Moreover, another advantage of the two-equation model estimation procedure carried out in this paper is that it allows us to observe both direct and indirect effects of the regressors on the probability of separating e-waste. It is important to remark that the preoccupation with environmental issues behaves as an endogenous factor, being a function of several exogenous regressors, which may affect the probability of separating e-waste in an indirect way. This is also very important information for policymakers seeking to select the priority groups for an e-waste recycling programme.

Thus, the results obtained from the two-equation binary model allow us to segment individuals in order to define adequately which users should be the focus of a specific campaign to encourage e-waste separation and collection because it allows policymakers to identify which factors directly and indirectly influence household behaviour. It is a proven fact that adopting measures tailored to the characteristics of the individual increases the effectiveness of e-waste management for two reasons: first, it will improve the level of knowledge of the environmental problems arising from the hazardous components of e-waste, and secondly, it will reinforce the conservation message embodied in these measures.

Specifically, the results obtained in the study point to two main ways of intervention: reinforcing environmental education (both from a formal and an informal approach) and reducing the costs of properly disposing of e-waste by improving the collection point networks.

Finally, we should note that this study has some limitations that open up interesting paths for future research. First, the empirical analysis has been carried out using cross-sectional data. Because past situations can influence future decisions, a next step of study could be to analyse the relationships between household e-waste attitudes and its drivers using time series or panel data. This kind of data can allow us to observe the evolution of the variables over time and to use other analytical procedures (e.g. unit root test, cointegration test) in line with other research developed in near areas such as CO_2_ emissions, as we can see in Lin and Xu (2020) and Xu and Lin (2018). Secondly, the study is focused on urban households from Spain. For this reason, an interesting next step of analysis would be to replicate our methodological purpose to other countries with different socio-economic and cultural characteristics. In this way, future research should also cover household e-waste separation and disposal in rural areas.

## Data Availability

The datasets analysed during the current study are available in the INE (2010) Survey on Households and the Environment 2008 microdata dataset (ftp://www.ine.es/temas/hogmed/datos_hogmed.zip).

## Appendix

For the case of households with/without members from 20 to 30 years old, the interaction variables allows to distinguish the following four categories:

• Group GL1_A: Household is in a *large city with* some members from 20 to 30 (*Large_city*=1, *M20to30*=1, and *Large_M20to30*=1).

• Group GS1_A: Household is in a *small city with* some members from 20 to 30 (*Large_city*=0, *M20to30*=1, and *Large_M20to30*=0).

• Group GL0_A: Household is in a *large city without* some members from 20 to 30 (*Large_city*=1, *M20to30*=0, and *Large_M20to30*=0).

• Group GS0_A: Household is in a *small city without* some members from 20 to 30 (*Large_city*=0, *M20to30*=0, and *Large_M20to30*=0).

If we want to obtain the average marginal effect of the city size in households with members between 20 and 30 years of age (age interval A) we must calculate:

$$ {\displaystyle \begin{array}{l}\Pr \left(\mathrm{GL}1\_\mathrm{A}\right)-\Pr \left(\mathrm{GS}1\_\mathrm{A}\right)=\Phi \left(\mathbf{x}\boldsymbol{\upbeta } \left| Large\_ city=1,M 20 to 30=1, Large\_M 20 to 30=1\right.\right)-\\ {}\Phi \left(\mathbf{x}\boldsymbol{\upbeta } \left| Large\_ city=0,M 20 to 30=1, Large\_M 20 to 30=0\right.\right)\end{array}} $$

However, if there is nobody in this age interval, the marginal effect will be:

$$ {\displaystyle \begin{array}{l}\mathit{\Pr}\left( GL0\_A\right)-\mathit{\Pr}\left( GS0\_A\right)=\Phi \left(\mathbf{x}\boldsymbol{\upbeta } \left| Large\_ city=1,M 20 to 30=0, Large\_M 20 to 30=0\right.\right)-\\ {}\Phi \left(\mathbf{x}\boldsymbol{\upbeta } \left| Large\_ city=0,M 20 to 30=0, Large\_M 20 to 30=0\right.\right)\end{array}} $$

Now we want to obtain the marginal effect of having people of interval A in the household against not having members of this age. In large cities, this partial effect will be calculated as:

$$ {\displaystyle \begin{array}{l}\mathit{\Pr}\left( GL1\_A\right)-\mathit{\Pr}\left( GL0\_A\right)=\Phi \left(\mathbf{x}\boldsymbol{\upbeta } \left| Large\_ city=1,M 20 to 30=1, Large\_M 20 to 30=1\right.\right)-\\ {}\Phi \left(\mathbf{x}\boldsymbol{\upbeta } \left| Large\_ city=1,M 20 to 30=0, Large\_M 20 to 30=0\right.\right)\end{array}} $$

However, in a small city this effect is given by:

$$ {\displaystyle \begin{array}{l}\mathit{\Pr}\left( GS1\_A\right)-\mathit{\Pr}\left( GS0\_A\right)=\Phi \left(\mathbf{x}\boldsymbol{\upbeta } \left| Large\_ city=0,M 20 to 30=1, Large\_M 20 to 30=1\right.\right)-\\ {}\Phi \left(\mathbf{x}\boldsymbol{\upbeta } \left| Large\_ city=0,M 20 to 30=0, Large\_M 20 to 30=0\right.\right)\end{array}} $$

Analogously, we obtain the marginal effects corresponding to the other interval age (B, C, and D), in order to obtain the values of the cells in Tables 7 and 8.

## Footnotes

1 The composition of e-waste varies widely, depending on the type and age of the equipment. However, e-waste is usually composed of metals (copper, iron, silver, gold, nickel, and tin, among others), plastic, and refractory oxides. For more details about e-waste composition and the potential associated contaminants, see Forti et al. (2020).

2 A broad analysis of the relevant literature on household recycling can be found in Hornik et al. (1995) and Saphores et al. (2006). In the specific field of e-waste, an in-depth literature review can be found in Pérez-Belis et al. (2015a).

3 For more details about the target characteristics of this survey, see INE (2009).

4 It is well known that one of the categories corresponding to the same factor will be not included in the model, being the “control group”.

5 The logistic cumulative distribution function is $\Lambda \left(\mathbf{x}\boldsymbol{\upbeta } \right)=\frac{e^{\mathbf{x}\boldsymbol{\upbeta }}}{1+{e}^{\mathbf{x}\boldsymbol{\upbeta }}}$.

6 The full expression of the log-likelihood function can be found in Wooldridge (2002).
